# Supplementary material for: MetaRibo-Seq measures translation in microbiomes
Source: Nat Commun. 2020 Jun 29;11:3268. doi: 10.1038/s41467-020-17081-z (PMC7324362; doi:10.1038/s41467-020-17081-z)
Supplement: Supplementary file 10 — Supplementary Data 7 [file 41467_2020_17081_MOESM10_ESM.zip › File2/Confidence_High_Taxonomy/320320_out.krona.html]

Javascript must be enabled to view this page.

members
magnitude
magnitudeUnassigned
count
unassigned
taxon
rank

320320\_out

3

superkingdom
2
3

phylum
976
3

200643
3
class

3
171549
order

3
171552
family

3
838
genus

species

SRS012849\_contig\_number\_24753SRS017307\_contig\_number\_32084SRS023914\_contig\_number\_10435
3
165179
